# First symptoms and health care pathways in hospitalized patients with acute heart failure: ICPS2 survey. A report from the Heart Failure Working Group (GICC) of the French Society of Cardiology

**DOI:** 10.1002/clc.23666

**Published:** 2021-06-26

**Authors:** Florence Beauvais, Lamia Tartière, Théo Pezel, Chloé Motet, Marie‐Claude Aumont, Guillaume Baudry, Jean‐Christophe Eicher, Michel Galinier, Barnabas Gellen, Julien Guihaire, Damien Legallois, Benoit Lequeux, Delphine Mika, Frederic Mouquet, Muriel Salvat, Charles Taieb, Florian Zorès, Emmanuelle Berthelot, Thibaud Damy

**Affiliations:** ^1^ Department of Cardiology CHU Lariboisière, APHP Paris France; ^2^ Department of Cardiology Hôpital Leon Berard Hyères France; ^3^ Faculty of Medicine University of Nantes Nantes France; ^4^ Department of Cardiology CHU Bichat, APHP Paris France; ^5^ HCL, Service Insuffisance cardiaque Hôpital Louis Pradel Bron France; ^6^ Department of Cardiology CHU Dijon Dijon France; ^7^ Department of Cardiology CHU Rangueil Toulouse France; ^8^ Department of Cardiology ELSAN ‐ Polyclinique de Poitiers Poitiers France; ^9^ Department of Cardiology, Hôpital Marie Lanelongue Groupe Hospitalier Paris Saint Joseph, Université Paris Saclay Le Plessis Robinson France; ^10^ Department of Cardiology CHU de Caen Caen France; ^11^ Department of Cardiology CHU Poitiers Poitiers France; ^12^ Inserm, UMR‐S 1180 Université Paris‐Saclay Chatenay‐Malabry France; ^13^ Department of Cardiology Hopital prive le Bois Lille France; ^14^ Department of Cardiology CHU de Grenoble Grenoble France; ^15^ EMMA Clinic Fontenay‐sous‐Bois France; ^16^ Groupe Médical Specialisé Strasbourg France; ^17^ Department of Cardiology Hopital Bicêtre Bicêtre France; ^18^ Department of Cardiology, Referral Center for Cardiac Amyloidosis and DHU ATVB CHU Henri Mondor, APHP Creteil France

**Keywords:** comorbidities, healthcare pathway, heart failure, hospitalization, risk factors, symptoms

## Abstract

**Background:**

Acute heart failure (AHF) is a common serious condition that contributes to about 5% of all emergency hospital admissions in Europe.

**Hypothesis:**

To assess the type and chronology of the first AHF symptoms before hospitalization and to examine the French healthcare system pathways before, during and after hospitalization.

**Material and Methods:**

A retrospective observational study including patients hospitalized for AHF

**Results:**

793 patients were included, 59.0% were men, 45.6% identified heart failure (HF) as the main cause of hospitalization; 36.0% were unaware of their HF. Mean age was 72.9 ± 14.5 years. The symptoms occurring the most before hospitalization were dyspnea (64.7%) and lower limb edema (27.7%). Prior to hospitalization, 47% had already experienced symptoms for 15 days; 32% of them for 2 months. Referral to hospital was made by the emergency medical assistance service (SAMU, 41.6%), a general practitioner (GP, 22.3%), a cardiologist (19.5%), or the patient (16.6%). The modality of referral depended more on symptom acuteness than on type of symptoms. A sudden onset of AHF symptoms led to making an emergency call or to spontaneously attending an emergency room (ER), whereas cardiologists were consulted when symptoms had already been present for over 15 days. Cardiologists referred more patients to cardiology departments and fewer patients to the ER than general practitioners or the SAMU.

**Conclusion:**

This study described the French healthcare system pathways before, during and after hospitalization AHF. AHF clinic network should be developed to provide adequate care for all HF patients and create awareness regarding AHF symptoms.

**Abbreviations**
AHF: acute heart failure
CHD: coronary heart disease
COPD: chronic obstructive pulmonary disease
ER: emergency room
GICC: French Heart Failure Working Group of the French Society of Cardiology
GP: general physician
HF: heart failure
ICU: intensive care unit
LVEF: left ventricular ejection fraction
MISP: Medical Information System Program
SAMU: (French) Emergency medical assistance service
SD: standard deviation

## 1 INTRODUCTION

Acute heart failure (AHF) is a public health problem that affects about 64.3 million individuals world‐wide and 15 million in Europe. It is an important reason for emergency room (ER) visits.^1^, ^2^, ^3^ In France, AHF affects over 1 million patients and causes numerous single and multiple hospitalizations.^4^ In 2014, 165 000 patients were hospitalized for HF.^5^ It is estimated that the number of these already frequent hospitalizations will increase even more in the future, with important consequences on public health costs and health system functioning, if no relevant measures are undertaken.^6^

To date, for France, almost no information is available about the pathway of patients with AHF prior or during hospitalization and about problems that they may encounter, from first symptom appearance to patient care.^7^

Therefore, we conducted a survey on hospitalized AHF patients to learn about health care pathways as experienced by the patients themselves, and about problems encountered prior to hospitalization.

## 2 MATERIAL AND METHODS

Between April 1, 2018, and September 30, 2018, we conducted a national multi‐center observational, transversal survey in France. The survey was performed according to the guidelines of the International Association for proper conduct in epidemiological research and conformed to local legal requirements for the conduct of this type of study.^8^ In Europe, no ethics committee approval for this type of investigation is required. Patient participation was free and questionnaires were anonymous.

All members of the French Heart Failure Working Group of the French Society of Cardiology (GICC) were invited to participate in this survey. Data were collected using a questionnaire generated by cardiologists and public health physicians. Patients had to be at least 18 years of age and hospitalized or had to be previously hospitalized for a first episode of AHF as the main diagnosis for admission within the last 12 months. AHF was defined by the presence of new or worsening symptoms of HF (pulmonary edema, acute decompensated heart failure, or cardiogenic shock), requiring initiation or intensification of treatment for HF, according to the published standardized definitions.^9^

Eligible subjects were identified using the Medical Information System Program (MISP) coding or during consultations. Notably, the MISP identified all patients hospitalized with AHF as the main diagnosis for admission using a dedicated specific MISP code during the period of the study. This identification was independent of the existence of follow‐up by a cardiologist or not, and regardless of the recruitment center. Therefore, although the identification request by the MISP was performed by cardiologists from our working group, all eligible patients during the period could be included.

Patients who agreed to participate in the study completed the survey and mailed it back anonymously. Questions were about patient demographics, medical history, cardiac failure characteristics, types of symptom prior to hospitalization, pathway of care (before, during, and after hospitalization), current use of medication and of non‐medical treatment, as well as how patients perceived their illness. All questions including in the survey were presented in the Appendix S1. Depending on the question, patients could provide one or several answers, thus prevalence could add up to more than 100%. The survey was generated in a way that patients were not geared towards a HF diagnosis.

## 3 THEORY/CALCULATION

Only data from patients who provided full information about their age and gender were included in the analyses. Continuous variables were expressed as mean ± standard deviation (SD) and categorical variables as frequency with percentage. Statistical analyses were performed using the R version 3.6.3 [R Foundation] for categorical and numerical variables. The *t* test was used and a p value <0.05 was considered significant.

## 4 RESULTS

Forty centers participated in the study; 7000 questionnaires were sent to patients; of those, 1044 (15%) were returned. Among the 1044 returned, 251 (24%) were discarded (patient deceased, no information provided or more than 50% of the information missing, gender or age missing). Overall, 793 questionnaires were considered suitable for the statistical analysis.

### 4.1 Patient characteristics

Patient characteristics are shown in Table 1. The majority of patients were male (59.3%). Mean age was 72.9 years; women (75.2 years) were older than men (71.1 years). Most patients were retired and city residents. Coronary disease was the main cause for HF. The most frequent comorbidities included hypertension, diabetes, chronic obstructive pulmonary disease (COPD), and chronic kidney disease. Mean left ventricular ejection fraction (LVEF) was 40%; however, only 180 patients provided this information.

**TABLE 1:** Patient characteristics

	Patients
	*N* = 793
**Age (mean ± SD); years**	72.9 ± 14.5
Male	71.7±
Female	75.2
**Male; (*n*, %)**	470 (59.3)
**City residents; (*n*, %)**	525 (70.7)
**Activity; (*n*, %)**	
Working	74 (9.5)
Retired	615 (78.6)
On sick leave	51 (6.5)
Unemployed	42 (5.4)
**Risk factors; (*n*, %)**	
Hypertension	366 (46.2)
Dyslipidemia	106 (13.4)
Diabetes	184 (23.2)
Active smoker	47 (5.9)
**Comorbidities; (*n*, %)**	
Renal failure	124 (15.6)
Dialysis	14 (1.8)
COPD	157 (19.8)
Sleep apnea	86 (10.8)
**Medical history; (*n*, %)**	
Angioplasty	248 (31.3)
Coronary bypass	130 (16.4)
Pacemaker	153 (19.3)
ICD	120 (15.1)
Valve surgery	143 (18.0)
**LVEF** ^a^ **; (*n*, %)**	72 (40.0)

Abbreviations: COPD, chronic obstructive pulmonary disease; ICD, implantable cardioverter defibrillator; LVEF, left ventricular ejectionfraction; SD, standard deviation.

^a^ Only 180 patients answered this question.

### 4.2 Patient care pathway

**FIGURE 1:**
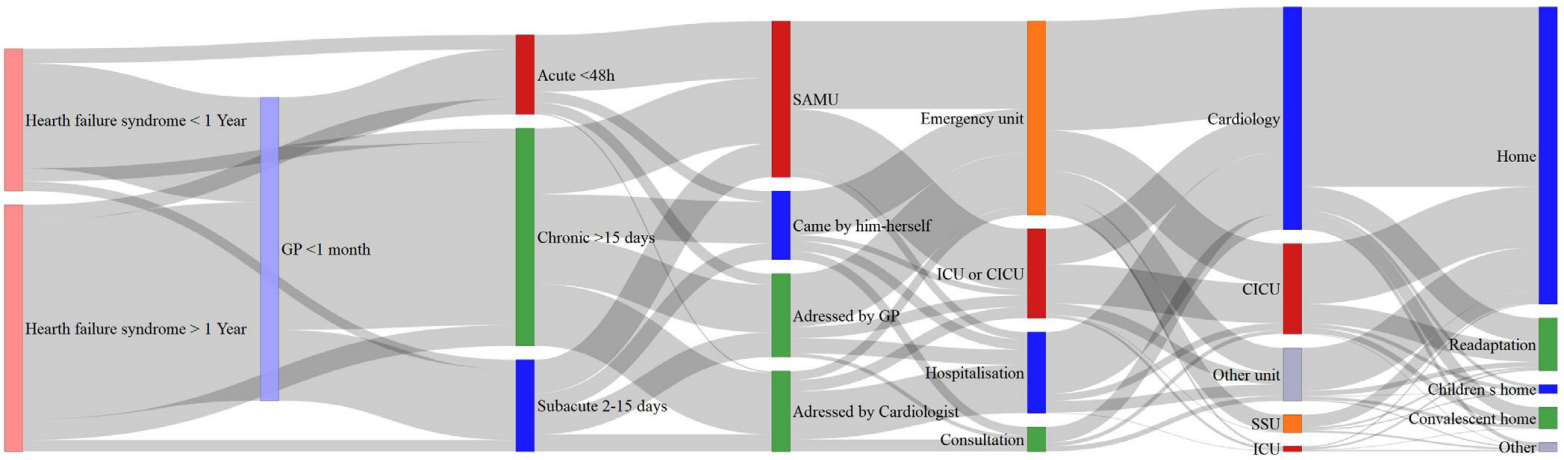
Patient care pathway, from the symptom‐appearing phase to the post‐hospital phase

Details about patient care pathway, from the symptom‐appearing phase to the post‐hospital phase, are provided in Figure 1.

### 4.3 Patient symptoms prior to hospitalization

Most patients were hospitalized due to dyspnea [68.0%) and/or lower limb edema (27.7%). Symptoms less often reported included chest pain (15.4%), fatigue (13.1%), tachycardia (12.8%), weight gain (10.0%), and other symptoms (16.7%). Dyspnea and lower limb edema were most often reported together (Table 2). Prior to hospitalization, symptoms had already been present for over 15 days in 47.4% of the patients, and for over 2 months in 32.3% of them, and 39.6% reported having seen their physician the month prior to admission.

**TABLE 2:** Symptom comorbidity

Dyspnea and							
Leg edema	Asthenia	Weight gain	Chest pain	Tachycardia	Other symptom(s)	Combination of other symptoms	
*n* (%)	*n* (%)	*n* (%)	*n* (%)	*n* (%)	*n* (%)	*n* (%)	p value
113 (21.8)	46 (8.9)	45 (8.7)	43 (8.3)	40 (7.7)	33 (6.4)	149 (28.7)	<0.00001

### 4.4 Hospital referral and admission

Appendix S1 provides detailed information about patient referral to the hospital, according to type of symptom and time of appearance. The emergency medical assistance service (SAMU) was the most common type of referral (41.6%). Only 19.5% of the patients were referred by their cardiologist. SAMU‐referred patients were older and more likely to suffer from acute coronary symptoms and chest pain, as well as dyspnea. Patients referred by a general physician (GP) or a cardiologist were more likely to suffer from lower limb edema. Other symptoms and recent weight gain in particular were less frequently reported by patients. Most patients referred by their GP or cardiologist had symptoms for over 15 days, including 76.8% of those referred by a cardiologist. Very few patients had consulted a cardiologist within 48 hours prior to hospitalization.

Patients were admitted to the hospital by different pathways, according to the type of referral and patient characteristics (Table 3). Non intensive care unit (ICU) was the most common way of recruitment (76.2%, including admission from ER department [48.0%], traditional cardiology units [19.6%] and consulting as out‐patients [8.6%]), whereas only 23.8% were recruited from ICU.

**TABLE 3:** Hospital admission by type of referral

	Patient referral				p value
	cardiologist	GP	Self‐admission	SAMU	
	*n* (%)	*n* (%)	*n* (%)	*n* (%)	
	142 (19.5)	162 (22.3)	121 (16.6)	303 (41.6)	
**Hospital admission**					
Hospital consultation	24 (17.5)	9 (5.7)	14 (11.8)	2 (0.7)	<0.0001
Emergency room	13 (9.5)	96 (60.8)	72 (60.5)	169 (56.1)	—
Non‐intensive care hospitalization	81 (59.1)	28 (17.7)	22 (18.5)	12 (4.0)	—
ICU or CICU	19 (13.9)	25 (15.8)	11 (9.2)	118 (39.2)	—

Abbreviations: CICU, cardiology intensive care unit; ICU, intensive care unit; GP, general practitioner; SAMU, emergency assistance medical service.

Most SAMU‐referred patients were admitted to ER or to ICU. Patients referred by their GP or attending the hospital on their own attended most often ER. The majority of cardiologist‐referred patients (59.1%) was directly admitted to a non‐intensive care unit and bypassed ER.

On average, patients admitted to ER or an ICU were aged 74.3 years and those admitted to a non‐intensive care unit 70.7 years. Patients admitted to an ICU were more often those with coronary heart disease (CHD) (46.8%) and were more likely to have chest pain, while those admitted to a non‐intensive care unit were more likely to have edemas. Appendix S1 specifies the unit of hospitalization according to symptoms. Other prior medical problems, risk factors or comorbidities did not appear to influence the type of hospital admission.

### 4.5 Care after admission

In total, 69.2% of the patients admitted to ER were transferred to a non‐intensive care unit. Those remaining were transferred to a department other than cardiology (Table 4).

**TABLE 4:** In‐patient department after hospital admission

Departments	Hospital admission			
	Hospital consultation *n* (%)	ER *n* (%)	Non‐intensive care hospitalization *n* (%)	ICU or CICU*n* (%)
	53 (7.3)	354 (48.7)	147 (20.2)	173 (24.3)
Cardiology	34 (70.8)	187 (55.2)	110 (76.9)	64 (39.3)
CICU	7 (14.6)	66 (19.5)	12 (8.4)	72 (44.2)
ICU	0 (0.0)	4 (1.2)	1 (0.7)	6 (3.7)
Other unit	7 (14.6)	58 (17.1)	20 (14.0)	20 (12.3)
UHS	0 (0.0)	24 (7.1)	0 (0.0)	1 (0.6)

Abbreviations: CICU, cardiology intensive care unit; ER, emergency room; ICU, intensive care unit; UHS, University Health Services.

### 4.6 Post‐hospitalization management

After being discharged, 76.2% of the patients returned back home. Only a minority was transferred to a cardiac rehabilitation ward (12.4%), to a convalescent home (6.4%), or went to live with a relative (2.1%). The youngest patients entered the cardiac rehabilitation program while the oldest ones were admitted to a convalescent home.

### 4.7 Patient disease perception and knowledge

Regarding the principal diagnosis for hospitalization, 45.6% of the patients believed it was HF, 18.4% arrhythmia, 10.1% a heart attack, 8.7% respiratory failure, and 7.9% pulmonary infection. Not considering the diagnosis being responsible for hospitalization, 64% of patients reported AHF, 23.2% respiratory failure, 2.7% venous insufficiency, and 7.0% did not know what their disease was. Most patients stated that the diagnosis was made by the hospital medical staff (51.1%) or their cardiologist (41.5%). Only 13.4% said that the diagnosis was made by their GP. A total of 22.6% of the patients provided a value for their left ventricular ejection fraction (LVEF); only a small number of patients provided information about medications. Thus, information about these 2 items was not considered for statistical analyses.

### 4.8 Patients respecting hygiene and diet advice

The prevalence of patients who reported having received, while being hospitalized, information about the need for a low‐salt diet was 63.7%, for treatment compliance 55.8%, for exercising 48%, and for monitoring their weight 43.5%. Only 31.6% of the patients reported that they had been able to follow this advice without help from others. Regular physical exercise and having a low‐salt diet were the two advices that were the most difficult to follow for 30.2% and 17.3% of the patients, respectively. Overall, 49% of the patients sought the help of a dietician at least once, and 24.4% of the patients searched for information on the internet.

## 5 DISCUSSION

This epidemiological study sheds some light on the health care pathways for AHF in France. It provides critical information about the symptoms that occur prior to hospitalization and the delay between symptoms and hospital admission, the types of hospital referral and hospital admissions, as well as post‐hospitalization management from a patient point of view.

Patients admitted to the hospital for AHF observed, prior to admission, major symptoms such as dyspnea or lower limb edemas. But they also reported other symptoms that were underestimated, such as weight gain, subsequent to congestion. Although already present for at least 2 weeks or more in over half of the patients, new symptom appearance resulted in only a small increase of consultations. Many AHF patients failed to recognize changes in weight as a potentially important indicator of clinical deterioration,^10^, ^11^ and many did not recognize or did not pay enough attention to the occurrence of new symptoms.^12^, ^13^ Reasons for HF patients not seeking immediate care have been detailed by Patel et al.^14^ Most often, patients believed that the problem was not serious enough and that it would disappear on its own, or importantly, were unsuccessful in making an appointment with their GP or their cardiologist. Moreover, HF symptoms might not have been identified by the physician, or treatment was not suitable. This probably emphasizes the need for increasing the use of remote monitoring solutions in order to early identify patient with increased dyspnea and/or lower limb edema or unusual symptoms in order to tailor treatment or suggest visit to GP or cardiologist and avoid hospitalization.

AHF diagnosis can be challenging because symptoms vary at presentation, and many different factors can cause an episode of acute heart failure. Thus, rapid identification of patients with AHF is the first step in providing effective care. In our study, referral was more influenced by the type and severity of recent symptoms rather than by symptom types only. Only chest pain, but not dyspnea of any severity, prompted rapid contact with emergency services. This suggests that in France, the ER, the SAMU and the GP are currently the first medical contact for most AHF patients prior to hospital admission.^15^ However, neither a GP nor a regular cardiologist played a major role in admitting the patient to hospital, and this was even more common when patients were not city residents. In the OFICA study, only about 10% of the patients hospitalized for AHF had consulted a cardiologist previously, and about half of the patients had not been referred by any of their regular practicians.^16^ Regarding hospital admission, almost 50% of patients were admitted to the ER, and about 20% bypassed the ER to attend a cardiology department directly. Cardiologists mostly referred patients with chronic symptoms. However, in the cases where the patient's cardiologist was involved, the patient most often bypassed the ER and received more specialized hospital care. Among the patients directly admitted to a cardiology department, those referred by their cardiologist were 3 times more numerous than those referred by their GP.

These results emphasize the difficulty for non‐cardiologists to obtain access to specialized care for their HF patients. New tools should be developed to ease communication between health care physicians in charge of heart failure patients and coordination with hospitals. Involving nurses in the process should also prompt earlier management of decompensated heart failure before hospitalization.

A recently published study by Gorliki et al. reported that a hospital course of care involving a cardiology department was associated with an increase in hospital survival in AHF patients. These finding may highlight the importance of collaboration between cardiologists and other in‐hospitals specialties, such as emergency physicians, in order to find the best in‐hospital pathway for patients with AHF.^7^

Access to specialized care has a strong impact on patient prognosis, including a shorter hospital stay and a lower risk of hospital death, with no specific difference, however, in outcome after hospital discharge.^7^, ^17^, ^18^ The heterogeneity of care pathway observed in this study and in that of Smeets et al. suggested the need for multiple solutions, specific to each situation.^19^ Collins et al. suggested that half of the patients admitted to the ER for HF could be safely discharged after a short observation stay.^20^ Most patients do not require acute medical care other than decongestion and few of them undergo invasive diagnostic tests or therapeutic procedures requiring intensive monitoring while hospitalized. Development of specialized medical care units which HF patients may attend as soon as symptoms occur may considerably decrease the number of hospital admissions and thereby reduce ER visits.

After hospital discharge, most patients returned home; only a minority was transferred to a cardiac rehabilitation unit, and about 75% of them had a follow‐up visit within 3 months following discharge. Similar results were observed in another French study.^21^ Interestingly, less than 50% of the patients knew that their hospitalization was due to AHF, and one third of the patients did not know that their illness was called HF. This lack of awareness has already been reported by Taylor et al.^22^

Our study emphasizes the key role of self‐care in patient management. Living with AHF can be challenging for most patients and may cause important lifestyle changes. Adherence to daily weight monitoring has been linked to a reduced risk of emergency department visits and hospitalizations for HF.^23^ Monitoring signs and symptoms of HF is particularly important and patients need to respond appropriately to any change in symptoms and seek medical assistance.^12^ More emphasis needs to be made on teaching HF patients and the public at large about the early warning signs for AHF, such as dyspnea, edema, weight gain, fatigue, etc. to ensure early diagnosis and treatment. Moreover, prescribing and organizing physical activity for patients is also essential.^24^ Thus, remote monitoring might increase patient awareness concerning symptoms.

AHF care pathways need to improve coordination between medical staff and patients and a multidisciplinary approach needs to be put in place. Establishing a French heart failure clinical network, such as those already existing in other European countries or in the USA may increase patients' and care givers' visibility and may help to improve early AHF patient care and to network between GPs, cardiologists and hospitals.^25^, ^26^, ^27^, ^28^ Several health care programs, such as PRADO, have been developed in France to improve AHF patient care after discharge from hospital.^29^ Another program involves registered nurses as key actors for identifying HF patients, informing them about their pathology, and carrying out remote monitoring, while networking with other health care professionals.^30^

We admit that this study has some limitations. First, the design of this retrospective study was based on the use of a survey, with a risk of selection bias. In addition, the use of a patient survey causes a risk of bias introduced by the survey instrument itself. Patients were mainly selected by cardiologists working in AHF clinics. Moreover, the patient return rate was low. However, we considered this rate adequate, considering the patient population mean age. Even though almost half of the patients in this study had consulted a physician during the month prior to hospitalization, we did not ask whether a AHF diagnosis had been made during this consultation.

Moreover, the majority of patients did not provide a LVEF value and data on medications could not be analyzed.

In conclusion, over the past decades, with a decreasing number of cardiologists in France, non‐cardiologists have difficulties in admitting their patients to specialized cardiology units. Both patients and the public need to be better informed about AHF symptoms. GPs, cardiologists, and hospitals need to network more efficiently to decrease ER admissions and, therefore, to improve patient prognosis.

## CONFLICT OF INTEREST

The authors have no conflict of interest to disclose.

## Supporting information


Appendix S1: Supporting information

## Data Availability

All data generated or analysed during this study are included in this published article [and its supplementary information files].

## References

[1] Ambrosy AP, Fonarow GC, Butler J, et al. The global health and economic burden of hospitalizations for heart failure: lessons learned from hospitalized heart failure registries. J Am Coll Cardiol. 2014;63(12):1123‐1133. 24491689 10.1016/j.jacc.2013.11.053

[2] Collins SP, Storrow AB. Moving toward comprehensive acute heart failure risk assessment in the emergency department: the importance of self‐care and shared decision making. JACC Heart Fail. 2013;1(4):273‐280. 24159563 10.1016/j.jchf.2013.05.002 PMC3804381

[3] Global, regional, and national incidence, prevalence, and years lived with disability for 354 diseases and injuries for 195 countries and territories, 1990–2017: a systematic analysis for the global burden of disease study 2017. Lancet. 2018;392(10159):1789‐1858. 30496104 10.1016/S0140-6736(18)32279-7 PMC6227754

[4] Tuppin P, Cuerq A, De Peretti C, et al. Two‐year outcome of patients after a first hospitalization for heart failure: a national observational study. Arch Cardiovasc Dis. 2014;107(3):158‐168. 24662470 10.1016/j.acvd.2014.01.012

[5] L'état de santé de la population en France, rapport 2017, 15 May 2020.

[6] Groenewegen A, Rutten FH. Sodium‐glucose co‐transporter 2 inhibitors and acute heart failure. Eur J Heart Fail. 2020;22(4):723‐725. 32072715 10.1002/ejhf.1759

[7] Gorlicki J, Boubaya M, Cottin Y, et al. Patient care pathways in acute heart failure and their impact on in‐hospital mortality, a French national prospective survey. Int J Cardiol Heart Vasc. 2020;26:100448. 31867437 10.1016/j.ijcha.2019.100448 PMC6906640

[8] Hoffmann W, Latza U, Baumeister SE, et al. Guidelines and recommendations for ensuring Good Epidemiological Practice (GEP): a guideline developed by the German Society for Epidemiology. Eur J Epidemiol. 2019;34(3):301‐317. 30830562 10.1007/s10654-019-00500-x PMC6447506

[9] Hicks KA, Tcheng JE, Bozkurt B, et al. 2014 ACC/AHA key data elements and definitions for cardiovascular endpoint events in clinical trials: a report of the American College of Cardiology/American Heart Association task force on clinical data standards (writing committee to develop cardiovascular endpoints data standards). J Am Coll Cardiol. 2015;66(4):403‐469. 25553722 10.1016/j.jacc.2014.12.018

[10] Gravely‐Witte S, Jurgens CY, Tamim H, Grace SL. Length of delay in seeking medical care by patients with heart failure symptoms and the role of symptom‐related factors: a narrative review. Eur J Heart Fail. 2010;12(10):1122‐1129. 20685686 10.1093/eurjhf/hfq122

[11] Carlson B, Riegel B, Moser DK. Self‐care abilities of patients with heart failure. Heart Lung. 2001;30(5):351‐359. 11604977 10.1067/mhl.2001.118611

[12] Schiff GD, Fung S, Speroff T, McNutt RA. Decompensated heart failure: symptoms, patterns of onset, and contributing factors. Am J Med. 2003;114(8):625‐630. 12798449 10.1016/s0002-9343(03)00132-3

[13] Goldberg RJ, Goldberg JH, Pruell S, et al. Delays in seeking medical care in hospitalized patients with decompensated heart failure. Am J Med. 2008;121(3):212‐218. 18328305 10.1016/j.amjmed.2007.10.032 PMC2377456

[14] Patel H, Shafazand M, Schaufelberger M, Ekman I. Reasons for seeking acute care in chronic heart failure. Eur J Heart Fail. 2007;9(6–7):702‐708. 17188930 10.1016/j.ejheart.2006.11.002

[15] Laveau F, Hammoudi N, Berthelot E, et al. Patient journey in decompensated heart failure: an analysis in departments of cardiology and geriatrics in the greater Paris university hospitals. Arch Cardiovasc Dis. 2017;110(1):42‐50. 28017276 10.1016/j.acvd.2016.05.009

[16] Logeart D, Isnard R, Resche‐Rigon M, et al. Current aspects of the spectrum of acute heart failure syndromes in a real‐life setting: the OFICA study. Eur J Heart Fail. 2013;15(4):465‐476. 23186936 10.1093/eurjhf/hfs189

[17] Kul S, Barbieri A, Milan E, Montag I, Vanhaecht K, Panella M. Effects of care pathways on the in‐hospital treatment of heart failure: a systematic review. BMC Cardiovasc Disord. 2012;12:81. 23009030 10.1186/1471-2261-12-81 PMC3507726

[18] Cluzol L, Cautela J, Michelet P, et al. Prehospital and in‐hospital course of care for patients with acute heart failure: features and impact on prognosis in "real life". Arch Cardiovasc Dis. 2017;110(2):72‐81. 27693052 10.1016/j.acvd.2016.05.004

[19] Smeets M, Van Roy S, Aertgeerts B, Vermandere M, Vaes B. Improving care for heart failure patients in primary care, GPs' perceptions: a qualitative evidence synthesis. BMJ Open. 2016;6(11):e013459. 10.1136/bmjopen-2016-013459 PMC5168518 27903565

[20] Collins SP, Pang PS, Fonarow GC, Yancy CW, Bonow RO, Gheorghiade M. Is hospital admission for heart failure really necessary?: the role of the emergency department and observation unit in preventing hospitalization and rehospitalization. J Am Coll Cardiol. 2013;61(2):121‐126. 23273288 10.1016/j.jacc.2012.08.1022 PMC3535319

[21] Tuppin P, Cuerq A, De Peretti C, et al. First hospitalization for heart failure in France in 2009: patient characteristics and 30‐day follow‐up. Arch Cardiovasc Dis. 2013;106(11):570‐585. 24140417 10.1016/j.acvd.2013.08.002

[22] Taylor CJ, Hobbs FD, Marshall T, Leyva‐Leon F, Gale N. From breathless to failure: symptom onset and diagnostic meaning in patients with heart failure‐a qualitative study. BMJ Open. 2017;7(3):e013648. 10.1136/bmjopen-2016-013648 PMC5353318 28283487

[23] Jones CD, Holmes GM, Dewalt DA, et al. Is adherence to weight monitoring or weight‐based diuretic self‐adjustment associated with fewer heart failure‐related emergency department visits or hospitalizations? J Card Fail. 2012;18(7):576‐584. 22748492 10.1016/j.cardfail.2012.05.004 PMC3389375

[24] Zores F, Iliou MC, Gellen B, et al. Physical activity for patients with heart failure: position paper from the heart failure (GICC) and cardiac rehabilitation (GERS‐P) working groups of the French Society of Cardiology. Arch Cardiovasc Dis. 2019;112(11):723‐731. 31542331 10.1016/j.acvd.2019.07.003

[25] Anguita Sánchez M, Lambert Rodríguez JL, Bover Freire R, et al. Classification and quality standards of heart failure units: scientific consensus of the Spanish Society of Cardiology. Rev Esp Cardiol. 2016;69(10):940‐950. 27576081 10.1016/j.rec.2016.06.006

[26] McDonagh TA, Blue L, Clark AL, et al. European Society of Cardiology Heart Failure Association Standards for delivering heart failure care. Eur J Heart Fail. 2011;13(3):235‐241. 21159794 10.1093/eurjhf/hfq221

[27] Task force of the Hellenic Heart Failure Clinics . How to develop a national heart failure clinics network: a consensus document of the Hellenic Heart Failure Association. ESC Heart Fail. 2020;7(1):15‐25. 32100972 10.1002/ehf2.12558 PMC7083479

[28] Hauptman PJ, Rich MW, Heidenreich PA, et al. The heart failure clinic: a consensus statement of the Heart Failure Society of America. J Card Fail. 2008;14(10):801‐815. 19041043 10.1016/j.cardfail.2008.10.005

[29] Améliorer la qualité du système de santé et maîtriser les dépenses: Propositions de l'Assurance maladie pour 2020, last access 15 May 2020

[30] Arrêté du 27 décembre 2019 relatif à l'autorisation du protocole de coopération « Télésurveillance, consultation de titration et consultation non programmée, avec ou sans télémédecine, des patients traités pour insuffisance cardiaque, par un infirmier, last access 15 May 2020

