# Supplementary material for: First symptoms and health care pathways in hospitalized patients with acute heart failure: ICPS2 survey. A report from the Heart Failure Working Group (GICC) of the French Society of Cardiology
Source: Clin Cardiol. 2021 Jun 26;44(8):1144–50. doi: 10.1002/clc.23666 (PMC8364729; doi:10.1002/clc.23666)
Supplement: Supplementary file 1 — Appendix S1: Supporting information [file CLC-44-1144-s001.docx]

**SUPPLEMENTARY FILES**

**SUPPLEMENTARY FILE 1**

1. **Survey of the study – English version**

**YOUR SITUATION TODAY**

**Thank you to specify:**

Your age: _ _ _

Your height in cm: _ _ _

Your weight in kg: _ _ _

Your gender:  Male  Female

Your Housing Department:

Do you live?  In a city  In the countryside

Do you live alone?  Yes  No

If not, do you live:  With your family  In an institution

**Among the following 4 propositions, check the proposition that most closely matches with your current status:**

 I work, I have a job  I am unemployed  I am retired  I am on sick leave

**Regarding your follow-up and your treatment, check it or the boxes that correspond to your situation today** (i.e. that is to say the day filling in the questionnaire)

 I am followed and treated for high blood pressure

 I am followed and treated for hypercholesterolemia

 I am followed and treated for renal failure

 I am being followed and treated for diabetes

 I am being followed and treated for sleep apnea syndrome

 I have stopped smoking

 I currently smoke

 I am currently on dialysis

 I am currently tired for no reason

 I am being followed and treated for chronic respiratory failure

 I have recently gained weight

 I am currently short of breath

**Regarding your medical and cardiovascular history, check the boxes that correspond to your situation**

• I benefited from the implantation of a stent (spring) in the arteries of the heart  Yes  No  I don't know

• I had cardiac surgery (coronary artery bypass grafts)  Yes  No  I don't know

• I had treatment for cancer (chemotherapy, surgery, radiotherapy)  Yes  No  I don't know

• I have a pacemaker  Yes  No  I don't know

• I have a defibrillator  Yes  No  I don't know

• I have had an operation on the cardiac valves by surgery or through the vessels  Yes  No  I don't know

About how your heart works

Have you ever had a heart ejection fraction measurement (LVEF by cardiac ultrasound or cardiac MRI or scintigraphy)? Yes No I don't know I don't understand

If yes, what was the first measure of LVEF: _ _ _ _% and in what year was this measure performed: _ _ _

Can you write the names of the medications you are taking today? (Copy your prescription):

| Drug name | Daily dose | Drug name | Daily dose |
| --- | --- | --- | --- |
|  |  |  |  |
|  |  |  |  |
|  |  |  |  |
|  |  |  |  |

**CONCERNING YOUR HOSPITALIZATION in emergency (or semi-emergency) that occurred during the year ...**

**What were the 2 main symptoms that led you to hospitalization? (1 or 2 possible answers)!**

 Shortness of breath

 Pain chest

 Rapid weight gain (in a few days)

 Palpitations (heart beating fast)

 Swelling of the legs, feet and / or stomach (edema)

 Fatigue for no reason

 Other symptoms, specify .............................................

**How long did this (these) symptom (s) appear before your hospitalization?** **(Only one answer)**

**Please specify the number, it is very important!**

A few months (at least 2 months), if yes how many: ...................... Months

A few weeks (between 2 weeks and 8 weeks), if yes how many: .................. Weeks

A few days (between 2 and 15 days), if yes how many: ........................Days

A few hours (between 1h and 48h), if yes how many: ..................... Hours

**Did you consult your doctor in the month preceding your hospitalization?**

Yes No

**During your hospitalization, how did you arrive at the hospital?** **(Only one answer possible)**

 I arrived by the SAMU or the fire brigade

 I saw my general practitioner who referred me to the hospital

 I saw my cardiologist who referred me to the hospital

 I came by myself because I was not feeling well

 I was brought in by a relative or family because I was not feeling well

**During your hospitalization, where did you arrive? (Only one answer possible)**

 I arrived directly in intensive cardiology care or intensive care

 I arrived in the emergency room and then I was hospitalized

 I arrived directly in the hospitalization department

 I had an appointment for a consultation or an exam and I was hospitalized because I was not well.

**Before your discharge from hospital, in which department have you been hospitalized?** **(Only one answer)**

 "Door" or emergency service

 Cardiological intensive care

 Resuscitation

 Internal or general medicine

 Cardiology

 Geriatrics

 Nephrology

 Pulmonology

 In another department: If other, please specify ......................... .................

**When you left the hospital, where did you go?** **(Only one answer)**

 I went home

 I went to my children’s house

 I went to a convalescent home

 I went to a cardiac rehabilitation service (or cardiovascular follow-up care)

 Other situation, specify ..........................................

**During your urgent hospitalization, the medical team must explain to you what was the main disease at the origin of your hospitalization. What is the main name that was used?** **(1 or 2 possible answers)**

 Myocardial infarction

 Arrhythmia

 Hypertension

 Lung infection

 Pulmonary embolism

 Venous insufficiency

 Aortic stenosis

 Hypertrophic heart disease

 Dilated heart disease

 Heart failure

 Renal failure

 Respiratory failure

 I was not given the diagnosis

 I didn’t understand or remember

 None of these words but another word has been used: specify which one: ................................. ..................

**CONCERNING THE ILLNESS for which you have been hospitalized ...**

**Giving your symptoms, what disease do you think you suffer from? (Many possible responses)**

 Respiratory failure

 Heart failure

 Another disease which: ..........................................................................................

 Venous insufficiency

 I do not know what is the diagnosis of my disease

**Who gave you the diagnosis of your disease?** **(Many possible responses)**

 No one

 The doctor during my hospitalization

 My general practitioner

 My cardiologist

 Another person, please specify which one: ........................................

**Since when do you think you have had symptoms of this disease?**

 Less than 1 year

 Between 1 and 3 years

 Between 3 and 5 years

 More than 5 years

**Which of the following symptoms is the FIRST symptom of your illness** (only one response)

**This symptom may be different or the same as the one for which you were hospitalized**

 Shortness of breath for light efforts

 Rapid weight gain (in a few days)

 Swelling of the legs, feet and / or stomach (Edema)

 Fatigue for no reason

 Others, specify ..............................................

 I had no symptoms before I was hospitalized

**What advice have you given to take better care of your disease?** **(Multiple answers possible)**

 Exercise or regular physical activity

 Regular weighing, watch your weight

 Adherence to treatment (I take my prescribed medication, I go to my medical appointments)

 Do not over-salt your food, eat little salt

 I wasn't given any advice.

**CONCERNING THE FOLLOW-UP of your illness for which you were hospitalized ...**

**If given, which of these rules was the most difficult to follow?** **(Only one answer)**

 Exercise or regular physical activity

 Regular weighing, watch your weight

 Adherence to treatment (I take my prescribed medication, I go to my medical appointments)

 Do not over-salt your food, Eat little salt.

**Which of these rules is (are) THE rule (s) you need help with?** **(Multiple answers possible)**

 Exercise or regular physical activity.

 Regular weighing (weight monitoring)

 Adherence to treatment (I take my prescribed medication, I go to my medical appointments)

 Do not over-salt your food.

 Eat little salt

 I don’t need any help, I’m doing everything very well

 I don't need any help because I won't follow these rules.

**CONCLUSION ... could you answer these last questions?**

• Have you ever seen a dietitian at least once? Yes No

• Have you ever had cardiac rehabilitation? Yes No

• Do you consult the Internet for information? Yes No

• Do you think that a smartphone / iPhone application would make it easier to manage your disease? Yes No

• Have you made contact with a patient association? Yes No

| **Do you have a question about your illness that you did not ask your doctor? Do not hesitate to ask... an answer will be provided and available in video on the site** [**www.giccardio.fr**](http://www.giccardio.fr)  ……………………..…………………..…………………..…………………..…………………..…………………..…………………..…………………..…………………..…………………..…………………..…………………..…………………..…………………..…………………..…………………..…………………..…………………..………………………..  Do not put any information that can identify you (no surname, no first name, no city of residence, no name of the doctor, no date of consultation) |
| --- |
| **You may have comments to make on your management, on how your disease was diagnosed... Do not hesitate to use this space!**  ……………………..…………………..…………………..…………………..…………………..…………………..…………………..…………………..…………………..…………………..…………………..…………………..…………………..…………………..…………………..…………………..…………………..…………………..……………………….. |

**Many thanks for your help and participation.**

**Your answers will allow us to improve the care of future hospitalized patients!**

**PLEASE SLIP THIS QUESTIONNAIRE IN THE ENVELOPE AND DROP IT IN A MAILBOX**

1. **Survey of the study – French version (original version)**


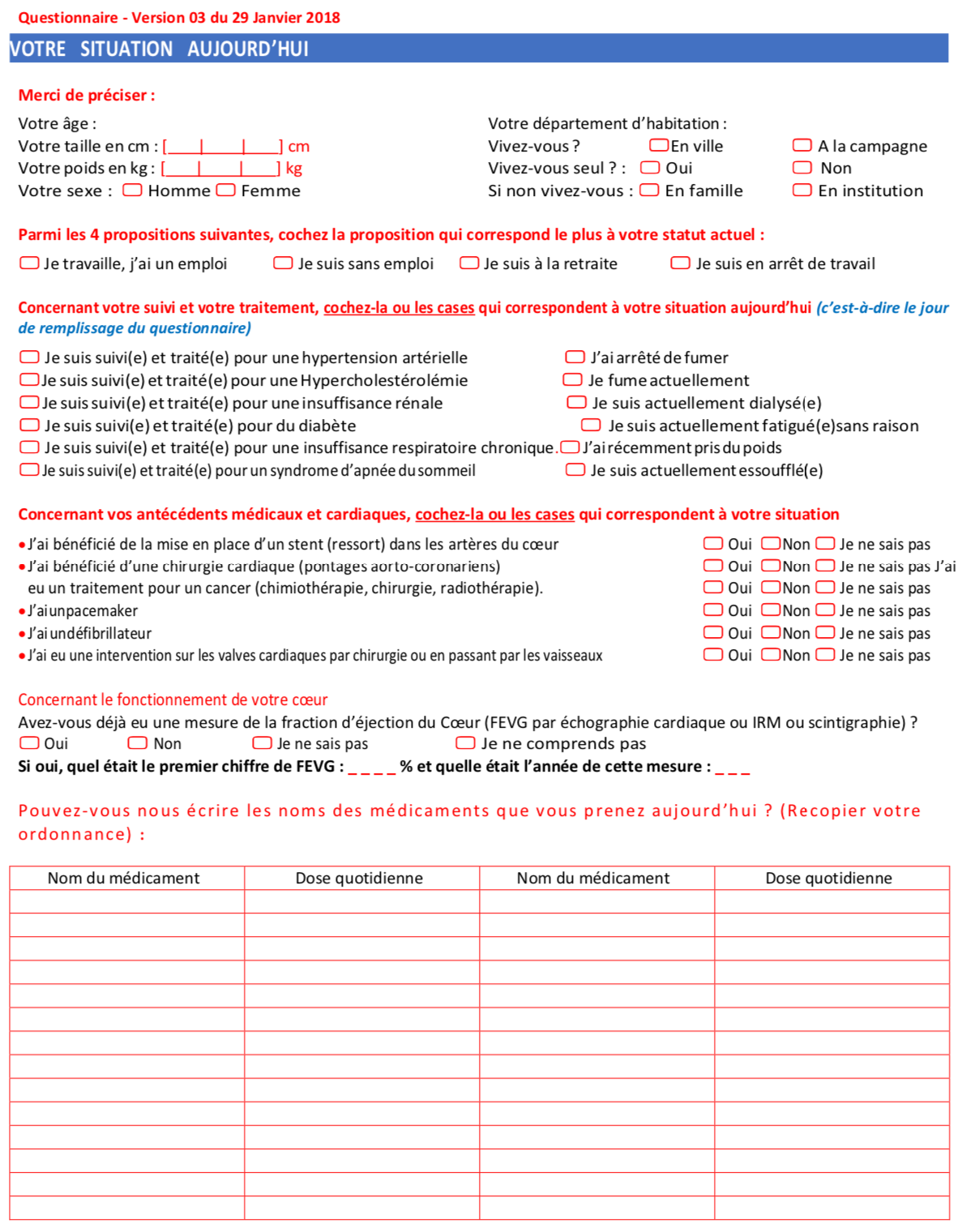


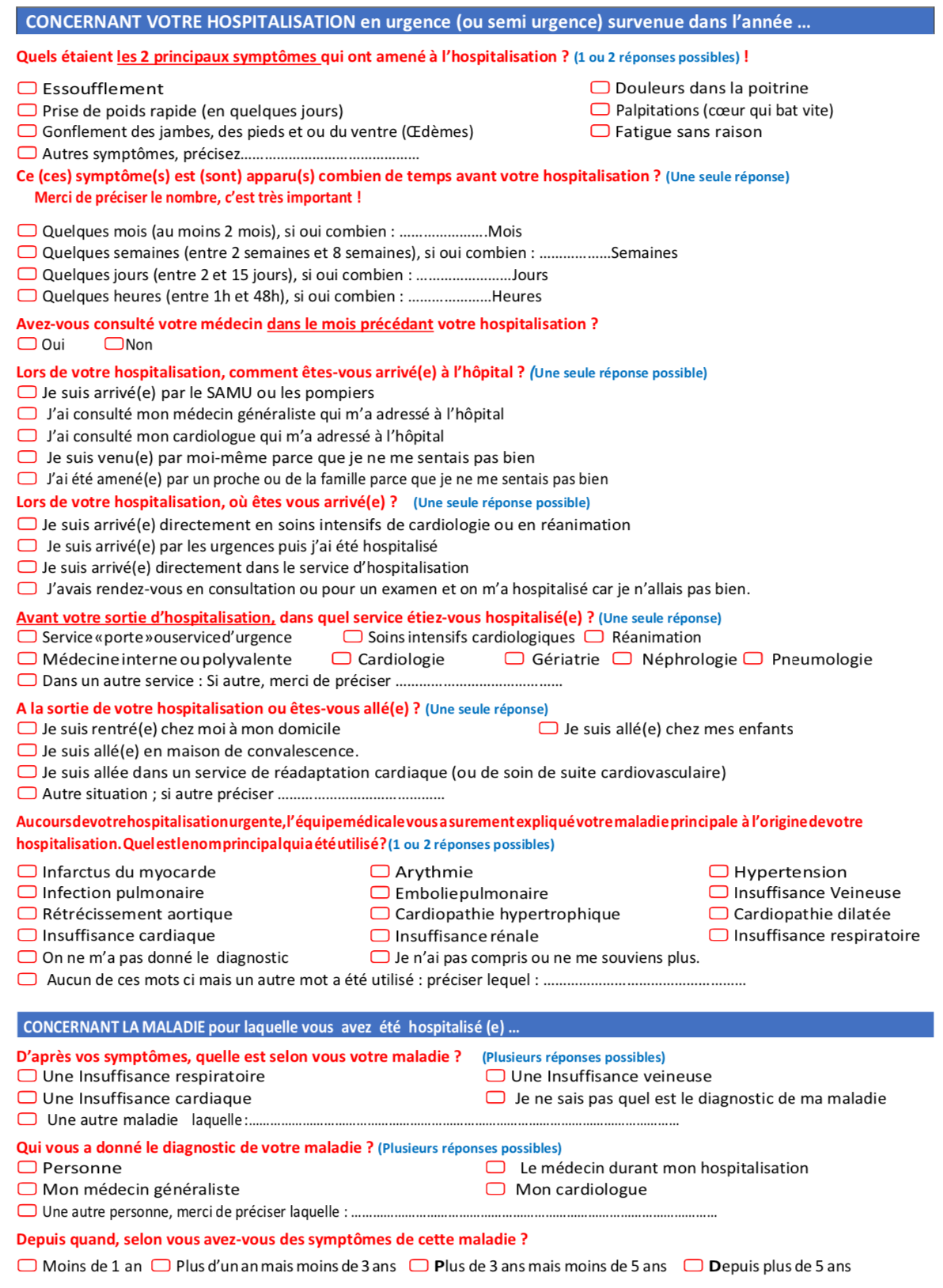


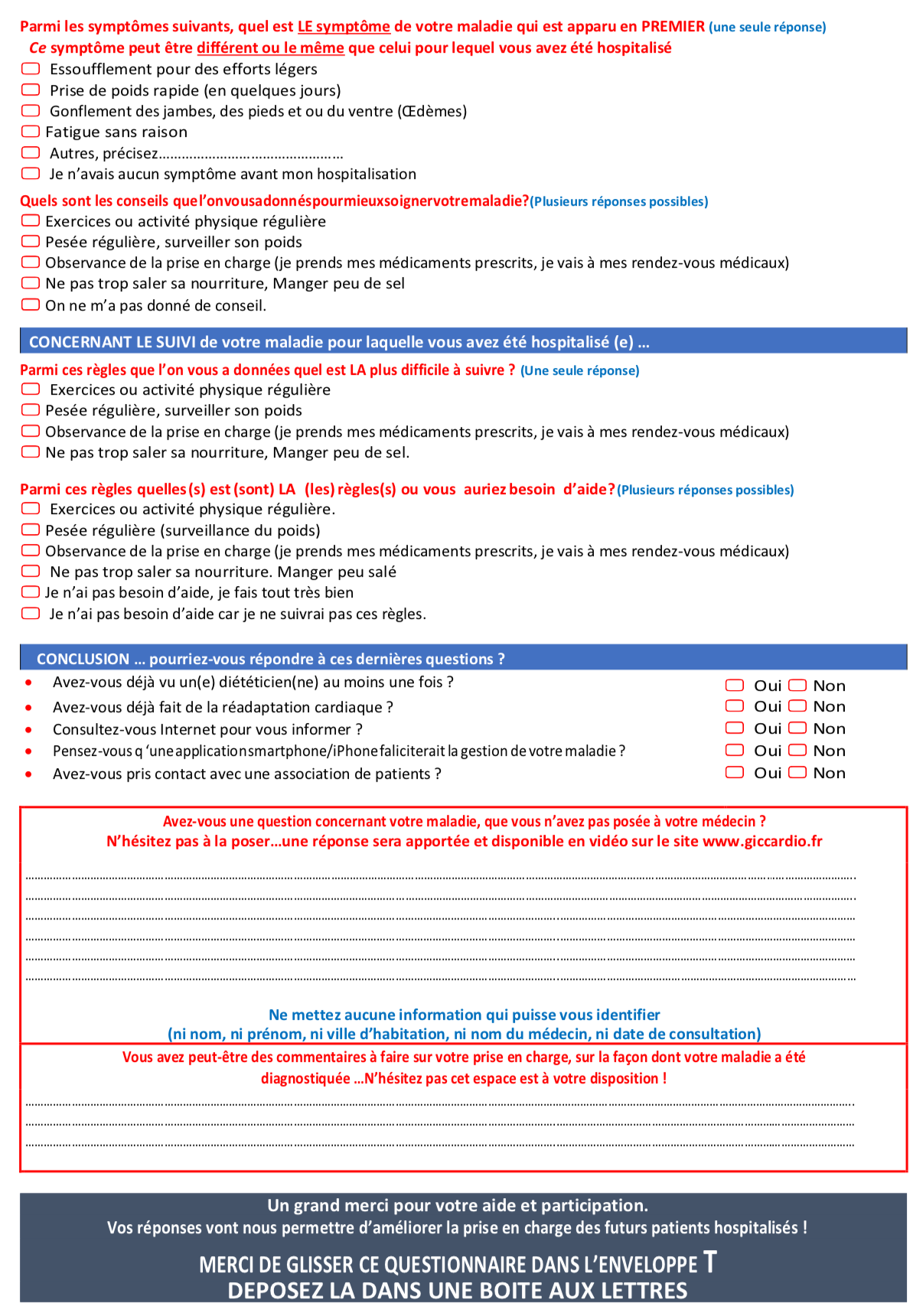


**SUPPLEMENTARY FILE 2**

**Table. Types of Referral to Hospital by Patient Symptoms and Symptom Occurrence**

|  | **Patient referred by** | | | | **pvalue** |
| --- | --- | --- | --- | --- | --- |
|  | **Cardiologist**  **n (%)**  **142 (19.5)** | **GP**  **n (%)**  **162 (22.3)** | **Self-admission**  **n (%)**  **121 (16.6)** | **SAMU**  **n (%)**  **303 (41.6)** |  |
| **Age(mean ±SD) (years)** | 70.7 ±12.9 | 73.8 ±13.5 | 71.0±15.2 | 75.2 ±13.6 | 0.0003 |
| **City dwellers** | 87(62.6) | 98(64.5) | 82(71.9) | 216(77.1) | 0.005 |
| **Male gender** | 97(68.3) | 91(56.2) | 80(66.1) | 171(56.4) | 0.035 |
| **Symptoms* before hospitalization** | | | | | |
| Dyspnea | 105 (73.9) | 116 (71.6) | 87 (71.9) | 192 (63.4) | 0.075 |
| Increased weight | 21 (14.8) | 18 (11.1) | 14 (11.6) | 20 (6.6) | 0.039 |
| Leg edema | 40 (28.2) | 62 (38.3) | 36 (29.8) | 64 (21.1) | 0.0013 |
| Chest pain | 8 (5.6) | 22 (3.6) | 18 (14.9) | 64 (21.1) | 0.0002 |
| Tachycardia | 26 (18.3) | 24 (14.8) | 15 (12.4) | 28 (9.2) | 0.045 |
| Fatigue | 15 (10.6) | 25 (15.4) | 20 (16.5) | 36 (11.9) | 0.38 |
| Other | 18 (12.7) | 18 (11.1) | 13 (10.7) | 74 (24.4) | 0.0001 |
| **Symptoms occurrence** | | | | | |
| Acute <48h | 3 (2.4) | 15 (10.6) | 16 (14.4) | 107 (39.6) | <0.0001 |
| Subacute 2-15 days | 26 (20.8) | 42 (29.8) | 34 (30.6) | 59 (21.9) | --- |
| Chronic>15 days | 96 (76.8) | 84 (59.6) | 61 (55.0) | 104 (38.5) | --- |

SD: standard deviation. GP: general practitioner. SAMU: emergency assistance medical service.

*: symptoms were self-reported.

**SUPPLEMENTARY FILE 3**

**Table. Hospital Admission by Type of Symptoms**

|  | **Hospital Admission** | | | | **p-value** |
| --- | --- | --- | --- | --- | --- |
|  | **Hospital Consultation**  **n (%)**  **53 (7.3)** | **ER**  **n (%)**  **354 (48.7)** | **Non-intensive care**  **Hospitalization n (%)**  **147 (20.2)** | **ICU or CICU n (%)**  **173 (24.3)** |  |
| **Symptoms* before hospitalization** | | | | | |
| Dyspnea | 35 (66.0) | 248 (70.1) | 106(72.1) | 111(64.2) | 0.40 |
| Increased weight | 5 (9.4) | 38 (10.7) | 18(12.2) | 13(7.5) | 0.53 |
| Leg edema | 21 (39.6) | 106 (29.9) | 37 (25.2) | 36 (20.8) | 0.026 |
| Chest pain | 5 (9.4) | 47 (13.3) | 15 (10.2) | 42 (24.3) | 0.0017 |
| Tachycardia | 5 (9.4) | 43(12.1) | 27(18.4) | 18(10.4) | 0.15 |
| Fatigue | 9(17.0) | 47(13.3) | 17(11.6) | 22(12.7) | 0.77 |
| Other | 5(9.4) | 61(17.2) | 21(14.3) | 38(22.0) | 0.13 |

ER: emergency room. ICU: intensive care unit. CICU: cardiology intensive care unit. P: p value. *: symptoms were self-reported.
